# Evaluating the association between the Mediterranean-DASH Intervention for Neurodegenerative Delay (MIND) diet, mental health, and cardio-metabolic risk factors among individuals with obesity

**DOI:** 10.1186/s12902-023-01284-8

**Published:** 2023-02-02

**Authors:** Abnoos Mokhtari Ardekani, Sahar Vahdat, Ali Hojati, Hadi Moradi, Ayda Zahiri Tousi, Farnoosh Ebrahimzadeh, Mahdieh Abbasalizad Farhangi

**Affiliations:** 1 Endocrinology and Metabolism Research Center, Institute of Basic and Clinical Physiology Science & Physiology Research Center, Kerman University of Medical Sciences, Kerman, Iran; 2 Isfahan Kidney Disease Research Center, Khorshid Hospital, School of Medicine, Isfahan University of Medical Sciences, Isfahan, Iran; 3 Department of Community Nutrition, Faculty of Nutrition, Tabriz University of Medical Sciences, Tabriz, Iran; 4 Faculty of Medicine, Belarusian State Medical University (BSMU), Minsk, Belarus; 5 Razavi Cancer Research Center, Razavi Hospital, Imam Reza International University, Mashhad, Iran; 6 Department of Internal Medicine, Faculty of Medicine, Mashhad University of Medical Sciences, Mashhad, Iran; 7 Tabriz Health Services Management Research Center, Tabriz University of Medical Sciences, Attar Neyshabouri St., Daneshgah Blvd., Tabriz, Iran

**Keywords:** MIND diet, Mental health, Cardio-metabolic risk, Diet

## Abstract

**Background:**

Several previous investigations have examined the brain-protective role of the Mediterranean-DASH Intervention for Neurodegenerative Delay (MIND) diet. However, more knowledge is needed about the MIND diet's other favorable impacts. The purpose of this study was to examine the relationship between the MIND diet, mental health, and metabolic markers in individuals with obesity.

**Methods:**

In this cross-sectional study, we included 339 individuals with obesity (BMI ≥ 30 kg/m^2^) aged 20–50 years. We utilized a semi-quantitative Food Frequency Questionnaire (FFQ), we assessed dietary intake, including 168 food items, and calculated the value of MIND. Metabolic syndrome (MetS) was defined according to the National Cholesterol Education Program Adult Treatment Panel III (NCEP-ATP III) guidelines. We assessed biochemical parameters using Enzymatic methods. Blood pressure and body composition were also determined.

**Results:**

Higher tertiles of the MIND diet score were associated with significantly higher energy intake, macronutrients, and brain-healthy food intakes (*P* < 0.001). Among the brain-unhealthy foods, only the intake of sweets and pastries was significantly lower in the highest versus lowest MIND tertiles. We also observed lower odds of stress (*P* < 0.05) and higher insulin sensitivity (*P* < 0.05) in the highest versus lowest MIND diet tertiles. We witnessed no significant changes in other parameters.

**Conclusion:**

Lower stress levels and higher insulin sensitivity independent of some confounders like age, BMI, sex, and physical activity were associated with the highest tertile of MIND diet score.

**Supplementary Information:**

The online version contains supplementary material available at 10.1186/s12902-023-01284-8.

## Background

As a major global epidemic, obesity is associated with many adverse health consequences, including cardiovascular events, type 2 diabetes mellitus, cancer, mental health problems, and higher mortality rates [1–5]. The increased prevalence of obesity has attracted public concern worldwide. As the World Health Organization (WHO) reported, more than 1.9 billion individuals over 18 were overweight and obese in 2016, and 13% of adults were obese overall. Accordingly, between 1975 and 2016, the global prevalence of obesity grew by approximately a factor of three [6–8]. The rise in obesity rates is attributed to inactivity and poor eating habits [9–12]. Similarly, obesity and overweight have been found to be prevalent in certain areas of Iran, as high as 76% [13].

Metabolic syndrome (MetS) is caused by obesity and is characterized by central obesity, abnormal serum lipids, glucose intolerance and insulin resistance, and hypertension [14]. The melanocortin system is a crucial neural mechanism that regulates body weight and may lead to numerous obesity-related health issues, including eating disorders, hypertension, type 2 diabetes [15–17]. Melanocortin receptors, agouti-related protein (AgRP), and melanocortin peptides (including α-MSH) are important components of the melanocortin system [18]. Of the five subtypes of melanocortin receptors (MC1-R to MC5-R), the Melanocortin 4-receptor (MC4-R) is most related to regulating body weight [19], and the activation of MC4-R reduces food intake and increases energy expenditure [20]. A natural peptide, agouti-related protein (AgRP), can inhibit the activity of the MC4-R [21, 22]. Accordingly, obese patients have higher circulating levels of AgRP, which may contribute to obesity development [23]. The anabolic effects of AgRP occur partially through competitive antagonism of α-melanocyte-stimulating hormone (α-MSH) at the melanocortin receptor (MC-R) [24]. Consequently, AgRP neurotransmission inhibits MC4-R activity, stimulates appetite, and leads to weight gain [25]. Also, α-MSH is a neuropeptide that regulates several physiological processes, such as energy homeostasis and food intake [26]. It has a crucial role in weight control because it stimulates the MC4-R, which reduces food consumption [25]. Evidence showed a positive association between plasma concentrations of α-MSH and visceral fat in obese males [27].

Diet is a modifiable risk factor of obesity and its related co-morbidities. Some recent studies evaluated the link between a healthy diet and disease outcomes [28, 29]. The majority of this research focused on the association between isolated dietary components (e.g., isolated effects of vitamins or minerals) [30–32], as well as the role of dietary patterns like the Mediterranean Diet or the Dietary Approaches to Stop Hypertension (DASH) [33–37] and dietary indices (e.g., glycemic and inflammatory) [38, 39] in developing obesity and metabolic disorders. The Mediterranean-DASH Intervention for Neurodegenerative Delay (MIND) diet was developed recently focusing on the neuro-protective effects of the Mediterranean diet and the DASH diet [40]. The MIND diet is mostly based on plant-based foods and encourages the high consumption of berries and green leafy vegetables, nuts, olive oil, whole grains, and beans. Meanwhile, this diet limits the intakes of animal-based foods and high saturated fat foods like red meat and its products, butter, sweets, and pastries [41].

Some previous studies assessed the brain–protective role of MIND and reported that slower cognitive decline, reduced rates of cognitive impairment, and reduced incidence of Alzheimer’s disease [42, 43] and Parkinson’s disease [44, 45] were associated with higher MIND diet score [42, 46]. Moreover, some recent studies evaluated other possible beneficial effects of MIND on cardiovascular diseases (CVD) mortality [40, 47, 48], central or general obesity [49], MetS and its components [50], or cardiac remodeling [51]. The results of these studies are inconsistent; while one of them reported improved blood pressure and reduced cardiac remodeling [51], the others reported no significant association between obesity measurements and biochemical risk factors of obesity in the general population [49, 50]. Accordingly, this study was designed to examine the potential relationship between the MIND diet and metabolic risk factors, including lipids profile, glycemic indicators, and mental health in individuals with obesity.

## Methods and materials

### Participants

**Fig. 1:**
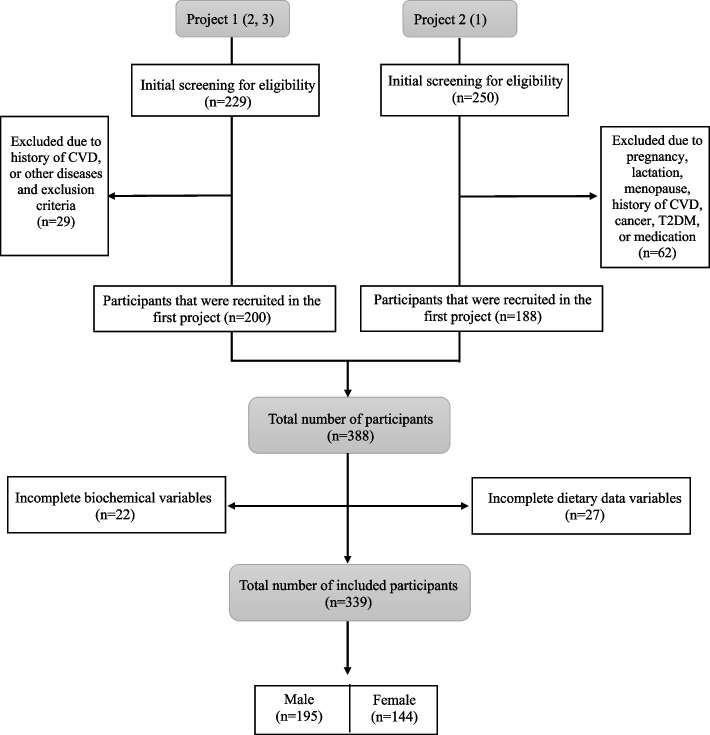
The subjects were selected from two projects conducted at Tabriz University of Medical Sciences

We included 339 obese people from Tabriz and Tehran, Iran, in our cross-sectional analysis. The subjects were drawn from two recent studies on obese individuals [52–54]. The study flowchart is displayed in Fig. 1. According to World Health Organization standards, a body mass index (BMI) of 30 kg/m2 or higher is considered obese [55]. Subjects in the 20–50 age group with a body mass index (BMI) of more than 30 kg/m2 were recruited through public announcements and enrolled in the study. We excluded all pregnant, lactating, and menopausal women. Also, other exclusion criteria were cardiovascular diseases (CVD), recent bariatric surgery, cancer, diabetes mellitus, hepatic and renal diseases, malabsorptive disorders, and the use of weight-altering drugs or supplements. Through one-on-one interviews, a certified nutritionist conducted all phases of recruiting and data collecting.

Regarding ethical considerations, all participants provided written knowledgeable consent, and the study concept was approved by the Ethics Committee of Tabriz University of Medical Sciences, Tabriz, Iran, approved the study concept (Registration number: IR.TBZMED.REC.1401.647).

### General characteristics and anthropometric assessments

Sociodemographic information, including sex (male/female), age (continuous), smoking status (current smoker/ former smoker/never smoked), education attainment (illiterate/elementary school/secondary school/diploma/bachelor's degree/master's degree or higher), marital status (single/married/divorced or widowed), medical history, occupation, and family size were obtained using a demographic questionnaire. Individual factors such as educational status, employment status, house ownership, and family size were used to assess the socioeconomic status (SES) score [54]. On a Likert scale of 0 to 5, illiteracy was deemed 0, less than a diploma 1, diploma and an associate degree 2, bachelor's degree 3, master's degree 4, and higher degrees considered 5. Similarly, men and women were asked to report their occupational position on a 5-point scale (housewife: 1, employee: 2, student: 3, self-employed: 4, and others: 5, for women) (unemployed: 1; laborer, farmer, and rancher: 2; others: 3; employee: 4; and self-employed: 5, for men). Subjects were assigned 1 (family size of 3), 2 (family size of 4–5), and 3 points based on their family size (family size of more than 6). They were also granted 2 points if they had a house and 1 point if they did not own a house. Lastly, each participant was assigned a score ranging from 0 to 15 points based on their SES.

We employed bioelectrical impedance analysis (BIA) to evaluate body composition (Tanita, BC-418 MA, Tokyo, Japan). The participants’ heights and weights were measured using a stadiometer mounted on the wall and a Seca scale, and the results were rounded to the nearest 0.5 cm and 0.1 kg for height and weight, respectively (Seca company, Hamburg, Germany). A concise version of the International Physical Activity Questionnaire (IPAQ) was used to measure physical activity levels [56, 57]. Each subject's waist circumference (WC) and hip circumference (HC) were measured to the nearest 0.1 cm using a tape measure at the midpoint between the lower costal margin and the iliac crest, respectively. In addition, waist circumference in relation to hip circumference (the waist-to-hip ratio) and body mass index were measured (BMI). The same arm's blood pressure was taken twice using a mercury sphygmomanometer after at least 15 min of rest, and the average result was used for analysis. MetS diagnostic criteria were established using the NCEP Adult Treatment Panel III (ATP III) recommendations [58].

#### Dietary assessments

An Iranian-adapted validated semi-quantitative Food Frequency Questionnaire (FFQ) with 168 items was used to collect information on diet [59]. A trained nutritionist asked participants to keep a food diary for a year, including what they ate and how much they ate each day, week, month, and year. The quantity and frequency of each food item were measured in grams using kitchen tools. Ultimately, daily food intakes were analyzed using the Nutritionist IV software (N Squared Computing, California, USA). Using the data collected, we were able to develop the MIND diet score. The MIND diet components were brain-healthy foods, including green leafy vegetables, other vegetables, berries, nuts, whole grains, fish, beans, poultry, olive oil, and wine. Meanwhile, brain-unhealthy foods included butter and margarine, cheese, red meat and processed meats, fast-fried foods, pastries, and sweets [43]. Since we did not have any data on wine consumption, we did not consider it for calculating the final score [49]. The MIND diet score was calculated by categorizing participants based on their tertile intake of the diet components. For brain-healthy foods, the individuals in the first, second, and third tertiles were given 0, 0.5, and 1 scores, respectively. Meanwhile, for brain-unhealthy foods, the scores of 1, 0.5, and 0 were assigned to individuals of the first, second, and third tertiles, respectively. The consumption of olive oil was scored as one if the individual recognized it as the main oil often used at home and as 0 otherwise. Finally, the total MIND diet score was computed by summing each food item’s scores, ranging from 0 to 14. Furthermore, the MIND diet score cut-off values were determined as 6.5 for the first tertile, 6.5–8 for the second, and > 8 for the third.

#### Mental health assessment

To assess mental health, we used the self-administered form of the 21-item Depression, Anxiety, and Stress Scale (DASS-21). The reliability and validity of this questionnaire among the Iranian population were confirmed in previous studies [60–62]. For each of the three scales of DASS-21 (depression, anxiety, and stress), there were seven items, and the responses were graded on a four-point Likert scale ranging from zero ("totally not relevant to me") to three ("very relevant"). A total score for each component was determined by summing the scores of relevant items. Based on the obtained final score of each component, the subjects were categorized into five subgroups normal, mild, moderate, severe, and extremely severe. Higher scores denoted a higher degree of mental disorder.

#### Biochemical assessment

Each participant gave a single morning venous blood sample (10 ml) after fasting for 12 h the night before, and the samples were centrifuged at 4500 rpm for 10 min at 4 °C to separate the serum and plasma. Until analysis, the samples were stored at -70° C. Using a commercially available kit, fasting blood sugar (FBS), triglyceride (TG), serum total cholesterol (TC), and high-density lipoprotein cholesterol (HDL-C) were measured (Pars Azmoon, Tehran, Iran). Further, low-density lipoprotein cholesterol (LDL-C) levels were determined using the Friedewald equation [63]. Insulin concentrations in serum were measured with ELISA test kits (Bioassay Technology Laboratory, Shanghai Korean Biotech, Shanghai City, China). Also, homeostatic model assessment for insulin resistance (HOMA-IR) and quantitative insulin-sensitivity check index (QUICKI) was calculated [64]. Plasma α-MSH and AgRP levels were measured using ELISA kits (Bioassay Technology Laboratory, China). The minimum detectable range for AgRP and α-MSH levels were 1.03 pg/ml and 5.07 ng/L, respectively.

### Statistical analyses

The statistical analysis was carried out with SPSS software, version 21.0, with a significance level of < 0.05. Statistical data were presented as frequency (%) for categorical variables and mean ± standard deviation (SD) for continuous variables. In order to examine the differences between discrete and continuous variables across the different tertiles of the MIND diet score, Chi-square and one-way analysis of variance (ANOVA) were used. The confounding factors were selected using the following criteria: the exposure was related to the confounder, the outcome was related to the confounder (or the confounding variable was a risk factor or a surrogate risk factor for the outcome), and the confounding variable was not an intermediary variable [65]. Thus, we selected age [66], sex [67], SES [68, 69], physical activity [70], BMI [71], educational level [72, 73], and energy intake [74, 75] as the confounding variables since they were associated with cardio-metabolic risk factors. Additionally, we used the same confounding variables for mental health, except for energy intake [76–78]. Multinomial logistic regression analysis was utilized to estimate ORs and 95% confidence intervals for the presence of cardio-metabolic risk factors and mental health problems across tertiles of the MIND diet score in crude and two multivariable-adjusted models.

## Results

The comparison of demographic, anthropometric, and mental health variables between subjects according to different MIND tertiles is given in Table 1. Participants' general characteristics were not significantly different except for age, which was higher in the higher tertiles (*P* = 0.049). Also, there was no significant difference in mental health variables except for stress (*P* = 0.038). In addition, after multivariable adjustment, no significant difference was observed. The comparison of mental health components among participants (Table 1) showed that subjects at the highest tertiles of the MIND diet score had lower depression, anxiety, and stress. However, this difference was only significant for the stress component of DASS-21. However, no significant difference was observed in mental health components after multivariable adjustment. Table 2 shows dietary energy and macronutrient intake among participants across MIND tertiles. There was a higher intake of total energy (*P* < 0.001), fat (*P* < 0.001), protein (*P* < 0.001), and carbohydrate (*P* < 0.001) among subjects in the higher tertiles of the MIND diet score. Except for fat (*P* = 0.071), the intakes of total energy (*P* = 0.002), protein (P0.001), and carbohydrate (*P* = 0.001) were significantly higher among subjects in the higher tertiles of the MIND diet score after adjustment for multiple variables. Higher brain-healthy food components were also observed in participants with the highest tertile of MIND diet score (*P* < 0.001; Table 3). Moreover, for brain-unhealthy food items, only the intake of pastries and sweets was lower in the highest tertiles of the MIND diet score (*P* = 0.002; Table 3). After applying multivariable adjustment for food components of the MIND diet, we witnessed that the subjects in the higher tertiles consumed more nuts (*P* = 0.049), green leafy vegetables (*P* < 0.001), other vegetables (*P* = 0.015), fish (*P* = 0.026), beans (*P* = 0.036), and poultry (*P* = 0.014), but consumed less cheese (*P* = 0.001) than those in the lower tertiles. Furthermore, the intakes of butter and margarine (*P* = 0.004) and pastries and sweets (*P* = 0.016) were lower in the highest tertile. The odds ratios (ORs) and 95% confidence interval (CI) for cardio-metabolic risk factors among tertiles of the MIND diet score in two models are represented in Table 4. There was no significant association between the MIND diet score and odds of cardio-metabolic risk factors in all models except for participants at the highest MIND diet score tertile who had 16% higher values of QUICKI compared with the lowest tertile [OR: 1.160 (1.031–1.924; *P* < 0.05)] in the model I. Table 5 represents the adjusted ORs and 95% CI for mental health factors across the MIND diet score tertiles. Participants in the highest tertile of the MIND diet score had lower odds of stress in crude (OR: 0.920; 95% CI: 0.851–0.994), the model I (OR: 0.916; 95% CI: 0.845–0.992), and model II (OR: 0.913; 95% CI: 0.842–0.990) analyses. Also, we observed no significant association between the MIND diet score tertiles and the odds of depression and anxiety.

**Table 1:** General characteristics of participants by MIND tertiles

**Variables**	**Tertiles of MIND, mean (SD)**					
	**1**^**st**^** tertile (*n* = 113)**	**2**^**nd**^** tertile (*n* = 113)**	**3**^**rd**^** tertile (*n* = 113)**	**Total**	***P*-value**	*P*-value*
**Age (year)**	38.85 (9.23)	41.01 (8.30)	41.71 (9.46)	40.57 (9.07)	**0.049**	-
**BMI (kg/m**^**2**^**)**	32.78 (4.80)	32.41 (4.59)	32.77 (5.14)	32.66 (4.85)	0.803	0.372
**Sex [male n (%)]**	61 (56.5)	68 (60.2)	66 (55.9)	195 (57.52)	0.919	-
**Education [≥ 12 y; n (%)]**	86 (76.7)	78 (69.6)	95 (83.6)	259 (76.40)	0.431	0.651
**SES score**	10.01 (2.41)	9.88 (2.77)	9.98 (2.36)	9.96 (2.51)	0.950	-
**FM (%)**	33.93 (9.74)	32.66 (7.91)	34.89 (9.55)	33.81 (9.13)	0.425	0..144
**FFM (%)**	61.75 (12.4)	62.37 (13.07)	62.78 (11.63)	62.26 (12.35)	0.894	0.534
**WC (cm)**	106.20 (9.80)	106.36 (9.11)	107.55 (10.05)	106.73 (9.66)	0.514	0.156
**WHR**	0.93 (0.08)	0.93 (0.07)	0.93 (0.07)	0.93 (0.08)	0.987	0.582
**Household size**	3.32 (0.93)	3.45 (0.98)	3.58 (1.01)	3.44 (0.97)	0.345	0.350
**PA (MET.min/week)**	1982.42 (2788.61)	1710.23 (2828.82)	2892.61 (3997.97)	2161.83 (3220.19)	0.120	-
**Depression [n (%)]**					0.506	0.489
*Normal*	80 (71.2)	87 (76.7)	87 (76.4)	254 (74.93)		
*Mild*	22 (19.2)	15(13.3)	18 (16.4)	55 (16.22)		
*Moderate*	11 (9.6)	11(10.0)	8 (7.3)	30 (8.85)		
**Anxiety [n (%)]**					0.582	0.516
*Normal*	74 (65.8)	77 (68.3)	84 (74.5)	235 (69.32)		
*Mild*	18 (16.4)	17 (15.0)	12 (10.9)	47 (13.87)		
*Moderate*	21 (17.8)	15 (13.3)	15 (12.7)	51 (15.04)		
*Severe*	0	4 (3.3)	0	4 (1.18)		
*Extremely severe*	0	0	2 (1.8)	2 (0.59)		
**Stress [n (%)]**					**0.038**	0.93
*Normal*	95 (84.9)	101 (90)	107 (94.5)	303 (89.38)		
*Mild*	11 (9.6)	9 (8.3)	6 (5.5)	26 (7.67)		
*Moderate*	7 (5.5)	3 (1.7)	0	10 (2.95)		

*Abbreviations*; *BMI* body mass index, *SES* socioeconomic status, *FM* fat mass, *FFM* fat free mass, *WC* waist circumference, *WHR* waist to hip ratio, *PA* physical activity; All data are expressed as mean (± SD). *P*-values derived from one-way ANOVA with Tukey’s post-hoc comparisons. *Anthropometric variables and mental health factors were adjusted for demographic characteristics including age, sex, socioeconomic status, and physical activity

**Table 2:** Dietary energy and macronutrients among participants by MIND tertiles

	**Tertiles of MIND, mean (SD)**				
	**1**^**st**^** tertile (*n* = 113)**	**2**^**nd**^** tertile (*n* = 113)**	**3**^**rd**^** tertile (*n* = 113)**	***P-*value**	*P-*value*
Energy (Kcal/d)	2646.00 (902.21)	3069.91 (1123.62)	3309.49 (1149.45)	** < 0.001**	**0.002**
Protein (g/d)	84.76 (29.09)	99.70 (35.50)	113.44 (40.21)	** < 0.001**	** < 0.001**
Fat (g/d)	92.02 (41.05)	101.82 (49.71)	107.27 (49.20)	** < 0.001**	0.071
Carbohydrate (g/d)	387.44 (139.12)	462.00 (170.43)	495.04 (180.95)	** < 0.001**	**0.001**

All data are expressed as mean (± SD). *P*-values derived from one-way ANOVA with Tukey’s post-hoc comparisons. *All variables were adjusted for age, sex, socioeconomic status, and physical activity

**Table 3:** Dietary intakes of MIND diet components among participants across tertiles of the MIND diet score

	**Tertiles of MIND, mean (SD)**				
**MIND diet score components**	**1**^**st**^** tertile)*n* = 113)**	**2**^**nd**^** tertile (*n* = 113)**	**3**^**rd**^** tertile (*n* = 113)**	***P-*value**	*P-*value*
***Brain-healthy foods***					
Berries (g/d)	3.07 (12.38)	5.62 (10.27)	8.44 (17.02)	**0.013**	0.257
Nuts (g/d)	9.50 (12.45)	22.86 (57.78)	21.44 (39.36)	**0.032**	**0.049**
Green leafy vegetables (g/d)	44.70 (44.62)	63.57 (48.31)	97.56 (75.70)	** < 0.001**	** < 0.001**
Other vegetables (g/d)	197.80 ( 158.56)	266.50 ( 159.08)	369.40 (282.86)	** < 0.001**	**0.015**
Olive oil (g/d)	0.78 (2.68)	2.43 (4.79)	3.38 (6.64)	** < 0.001**	0.068
Whole grains (g/d)	129.65 (102.42)	172.58 (117.36)	193.00 (118.29)	** < 0.001**	0.262
Fish (g/d)	5.68 (9.48)	9.50 (13.24)	15.00 (15.94)	** < 0.001**	**0.026**
Beans (g/d)	39.37 (37.17)	58.46 (65.59)	72.98 (60.02)	** < 0.001**	**0.036**
Poultry (g/d)	21.16 (19.27)	31.04 (34.17)	34.66 (28.77)	** < 0.001**	**0.014**
***Brain unhealthy foods***					
Butter/ margarines (g/d)	5.86 (8.28)	3.42 (4.82)	4.56 (9.89)	0.077	**0.004**
Cheese (g/d)	32.52 (20.71)	28.37 (24.31)	26.05 (21.65)	0.091	**0.001**
Red meats and products (g/d)	32.86 (32.61)	29.65 (29.19)	29.19 (28.93)	0.617	0.459
Fast fried foods (g/d)	25.60 (36.80)	22.46 (34.33)	23.90 (54.44)	0.863	0.829
Pastries and sweets (g/d)	54.64 (42.06)	57.88 (43.07)	40.56 (32.44)	**0.002**	**0.016**

All data are expressed as mean (± SD). *P*-values derived from one-way ANOVA with Tukey’s post-hoc comparisons. *All variables were adjusted for age, sex, socioeconomic status, physical activity, and energy intake

**Table 4:** Crude and multivariable adjusted ORs and 95% CIs for cardio-metabolic risk factors across tertiles of the MIND diet score

Variables	Tertiles of MIND diet score								
	**Crude ORs (95% CI)**			**Model I**^**a**^** ORs (95% CI)**			**Model II**^**b**^** ORs (95% CI)**		
	**1**^**st**^**)*n* = 113)**	**2**^**nd**^** (*n* = 113)**	**3**^**rd**^** (*n* = 113)**	**1**^**st**^**)*n* = 113)**	**2**^**nd**^** (*n* = 113)**	**3**^**rd**^** (*n* = 113)**	**1**^**st**^**)*n* = 113)**	**2**^**nd**^** (*n* = 113)**	**3**^**rd**^** (*n* = 113)**
SBP (mmHg)	1	1.005 (0.971–1.041)	1.012 (0.975–1.051)	1	1.010 (0.981–1.039)	1.018 (0.989–1.047)	1	1.009 (0.980–1.039)	1.017 (0.987–1.048)
DBP (mmHg)	1	0.991 (0.947–1.038)	1.014 (0.967–1.063)	1	0.997 (0.964–1.031)	1.014 (0.980–1.049)	1	0.998 (0.964–1.033)	1.015 (0.980–1.051)
FBS (mg/dl)	1	1.008 (0.966–1.053)	1.012 (0.969–1.058)	1	1.005 (0.986–1.025)	1.010 (0.991–1.029)	1	1.004 (0.985–1.024)	1.009 (0.990–1.027)
TC (mg/dL)	1	0.999 (0.988–1.012)	0.996 (0.984–1.009)	1	0.997 (0.986–1.007)	0.997 (0.986–1.008)	1	0.997 (0.986–1.008)	0.997 (0.986–1.008)
LDL-C (mg/dL)	1	1.000 (0.991–1.008)	1.001 (0.992–1.009)	1	0.998 (0.986–1.009)	0.996 (0.984–1.008)	1	0.998 (0.987–1.010)	0.996 (0.984–1.009)
HDL-C (mg/dL)	1	0.958 (0.914–1.004)	0.982 (0.936–1.031)	1	0.970 (0.930–1.011)	0.983 (0.943–1.026)	1	0.968 (0.928–1.010)	0.981 (0.939–1.025)
TG (mg/dL)	1	0.998 (0.990–1.005)	1.000 (0.992–1.007)	1	1.001 (0.994–1.007)	1.002 (0.996–1.009)	1	1.000 (0.994–1.007)	1.002 (0.996–1.009)
Insulin (µIU/mL)	1	1.001 (0.772–1.299)	1.002 (0.765–1.312)	1	1.020 (0.981–1.060)	0.992 (0.951–1.035)	1	1.024 (0.984–1.063)	0.996 (0.953–1.040)
HOMA-IR	1	1.229 (0.482–3.136)	1.199 (0.765–1.312)	1	1.087 (0.943–1.254)	1.020 (0.872–1.194)	1	1.096 (0.948–1.267)	1.032 (0.878–1.213)
QUICKI	1	1.070 (0.591–3.98)	1.112 (0.714–1.463)	1	1.150 (0.691–4.933)	**1.160 (1.031–1.924)** ^*^	1	1.232 (0.787–4.201)	1.287 (0.689–1.967)
AgRP (pg/ml)	1	0.955 (0.906–1.007)	0.958 (0.907–1.011)	1	0.988 (0.969–1.007)	0.984 (0.964–1.005)	1	0.986 (0.966–1.005)	0.981 (0.961–1.003)
α-MSH (ng/l)	1	1.004 (0.998–1.010)	1.003 (0.997–1.009)	1	0.999 (0.997–1.001)	0.999 (0.997–1.001)	1	0.999 (0.997–1.001)	0.998 (0.996–1.001)

*Abbreviations*; *SBP* systolic blood pressure, *DBP* diastolic blood pressure, *FBS* fasting blood sugar, *TC* total cholesterol, *HDL* high density lipoprotein cholesterol, *LDL* low density lipoprotein cholesterol, *TG* triglyceride, *HOMA-IR* homeostatic model assessment for insulin resistance, *QUICKI* quantitative insulin-sensitivity check index, *AgRP* Agouti-Related Protein, *α-MSH* α-Melanocyte-stimulating hormone

^*^P < 0.05; ^a^ Model I: adjusted for age, sex, BMI, physical activity, socioeconomic status, and education level; ^b^ Model II: additionally adjusted for energy intake

**Table 5:** Crude and multivariable adjusted ORs and 95% CIs for mental health variables across tertiles of the MIND diet score

Variables	Tertiles of MIND diet score								
	**Crude ORs (95% CI)**			**Model I**^**a**^** ORs (95% CI)**			**Model II**^**b**^** ORs (95% CI)**		
	**1**^**st**^**)*n* = 113)**	**2**^**nd**^** (*n* = 113)**	**3**^**rd**^** (*n* = 113)**	**1**^**st**^**)*n* = 113)**	**2**^**nd**^** (*n* = 113)**	**3**^**rd**^** (*n* = 113)**	**1**^**st**^**)*n* = 113)**	**2**^**nd**^** (*n* = 113)**	**3**^**rd**^** (*n* = 113)**
Depression	1	0.983 (0.916–1.055)	0.955 (0.887–1.029)	1	0.988 (0.919–1.062)	0.954 (0.883–1.030)	1	0.986 (0.916–1.062)	0.951 (0.880–1.029)
Anxiety	1	0.983 (0.901–1.072)	0.949 (0.865–1.041)	1	0.988 (0.904–1.080)	0.949 (0.864–1.043)	1	0.983 (0.898–1.076)	0.948 (0.862–1.041)
Stress	1	0.948 (0.880–1.022)	0.920 (0.851–0.994)*	1	0.950 (0.879–1.027)	0.916 (0.845–0.992)*	1	0.951 (0.879–1.029)	0.913 (0.842–0.990)*

^*^P < 0.05, ^a^ Model I: adjusted for age, sex, BMI, and physical activity; ^b^ Model II: additionally adjusted for SES and education level

## Discussion

Our study examined the association between the MIND diet score and metabolic, demographic, and mental health variables among individuals with obesity. According to our findings, the highest tertile of the MIND diet score was significantly associated with lower odds of stress. However, the MIND diet score was not associated with cardio-metabolic risk factors except for QUICKI. We did not find any statistically significant differences in anthropometric variables between the tertiles of the MIND diet score. Due to the paucity of research on the relationship between the MIND diet score and metabolic or anthropometric parameters, making a scientific judgment about the results is challenging.

In our study, individuals with greater tertiles of the MIND diet had lower stress due to the neuro-protective effects of the MIND diet, similar to the findings reported by Koch M et al*.* [42]. The MIND diet, with a high intake of vegetables, green leafy vegetables, and nuts, has brain protective effects and can reduce the risk of Alzheimer’s disease [42], dementia [79], and cognitive decline [80]. MIND diet adherence lowers the risk of anxiety, depression, and other psychological disorders among the general population [81]. Also, due to the antioxidant and anti-inflammatory nature of the MIND diet and a lower intake of unhealthy food products such as red meat and sweets, the MIND diet can improve brain health. A cross-sectional study conducted by Aminifar A et al*.* [49] found no significant association between the odds of general and central obesity and adherence to the MIND diet in the general population with a BMI of 24.9 ± 3.8 kg/m2. In another study by Mahmoudpour S et al*.* [50], no significant difference in BMI was observed in different tertiles of the MIND diet, but in the logistic model, being at the highest MIND diet tertile was related to increased risk of obesity [OR: 1.19; CI: 0.80–1.78, *P* = 0.02]. Also, no significant difference in biochemical variables, including FBS, serum lipids, insulin, and blood pressure was observed between different MIND diet tertiles in our study, which is consistent with the results of the study by Mahmoudpour S et al. [50].

As previously stated, the MIND diet combines the DASH and Mediterranean diets. The positive effects of DASH or Mediterranean diet against obesity and metabolic disorders have been approved previously. For example, high adherence to the DASH diet is associated with reduced obesity prevalence [82, 83] and improved lipid profile [82, 84]. This is true for the positive effects of the Mediterranean diet alone [36, 85]. However, some studies reported conflicting results. For example, Tiong XT et al*.* [86] reported country-specific differences in the connections between the DASH diet and risk factors of cardio-metabolic diseases; adherence to the DASH diet was related to better lipid profile and cardio-metabolic risk factors among the Philippines. However, in the Malaysian adults, no significant connection was seen. The authors suggested a ‘need for country-specific tailoring of dietary interventions’ to observe their beneficial effects. Another study suggested considering gender differences in interpreting inconsistent results through the effects of the Mediterranean diet against metabolic disorders [87]. Although the most important reason for the observed discrepancies between the health benefits of MIND and DASH or Mediterranean diet is related to the difference in their components, dairy products are only limited to cheese, and others are excluded in the MIND diet. Also, fruits are almost excluded, and only berries are considered.

**Fig. 2:**
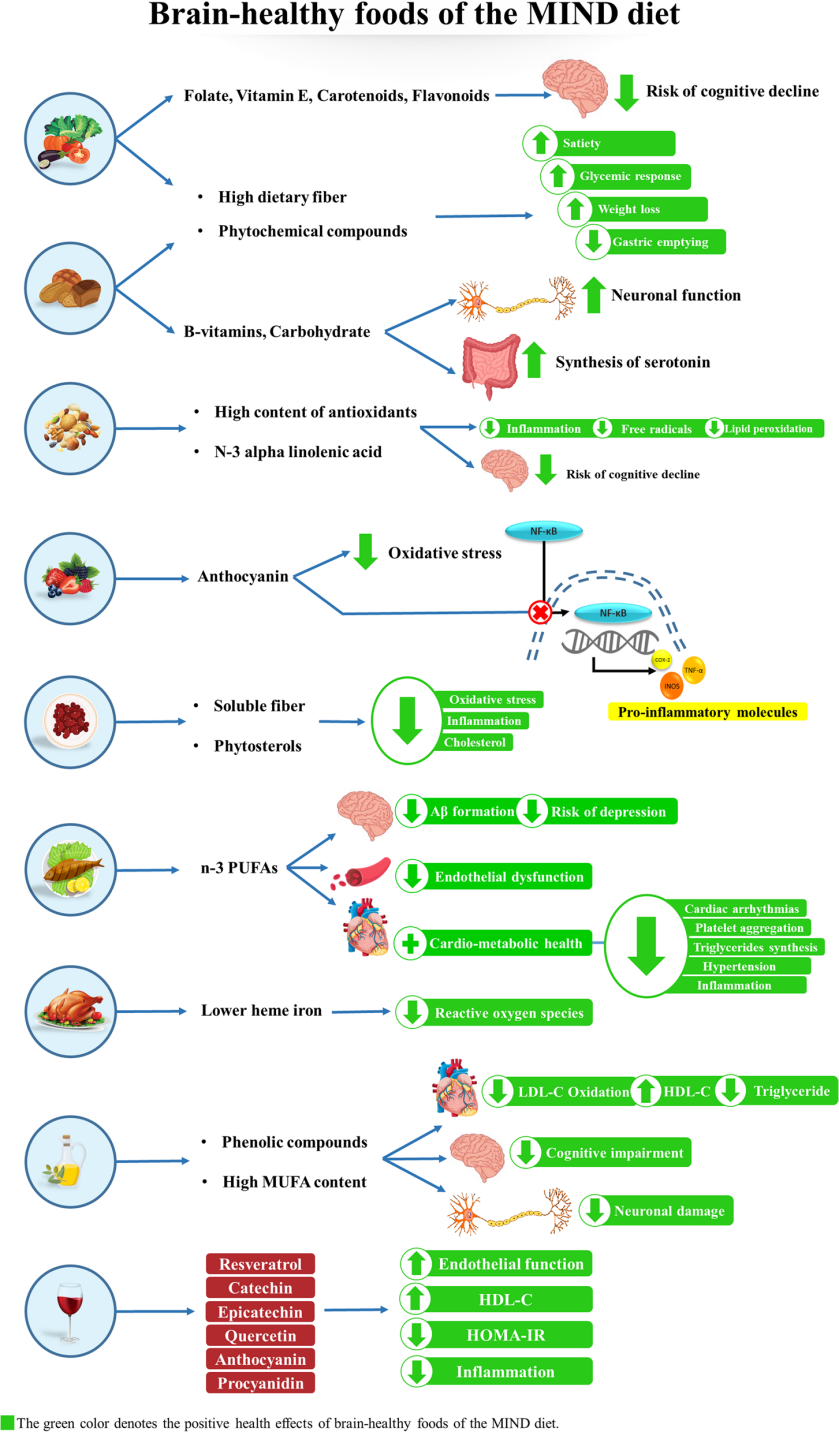
The possible underlying mechanisms for the health-improving effects of brain-healthy foods on mental health and cardio-metabolic risk factors

Our study also found a significant inverse association between odds of cardio-metabolic risk factors, including SBP, DBP, TC, and TG, with non-berry fruit consumption in the second tertile in crude analysis. After multivariate adjustment in both models, DBP had a significant inverse association with non-berry fruit consumption in the second tertile (Table S1). Although there was no significant association between tertiles of low-fat dairy consumption and cardio-metabolic risk factors (Table S2), we observed a significant inverse association between DBP and high-fat dairy consumption in the second tertile in crude analysis. Also, we found a significant positive association between high-fat dairy consumption and odds of HDL in crude analysis. In addition, there was a significant inverse association between high-fat dairy intake and odds of TG in multivariate-adjusted models (Table S3). According to these results, dairy and fruit consumption can be effective in improving cardio-metabolic risk factors. In this regard, previous studies showed that consuming dairy products is associated with reduced blood pressure [88] and improved lipid profile [89]; high calcium amount in dairy products prevents fat accumulation due to the uncoupled protein (UCP2) expression [90]. Also, fruits are a rich source of dietary polyphenols and antioxidants, and their health benefits in reducing CVD risk have been confirmed [91–93]. So, despite the efficacy of the MIND diet in maintaining a healthy brain, the MIND diet score may not be capable of predicting other obesity-related chronic conditions like cardio-metabolic diseases due to the exclusion of dairy products and non-berry fruits [49, 50]; however, further investigations are needed to confirm or decline this hypothesis. The possible underlying mechanisms for the effects of brain-healthy foods on mental health and cardio-metabolic risk factors are shown in Fig. 2.

Some studies on the variables affecting food preference indicated the significant effect of sociocultural factors on the selection of food variety worldwide [94]. A meal choice can be determined by cultural variables such as social norms and food accessibility. Certain meals are consumed habitually differently, depending on cultural factors [95]. Haghighian Roudsari et al*.* [94] demonstrated that psychological, social, and cultural factors affect the dietary preferences of Iranian people. In this regard, Sobhani et al*.* [96], according to Iranians' food habits, suggested that it is necessary to increase the consumption of fruits, vegetables, poultry, dairy, cereals, and legumes and to decrease the consumption of red meat, rice, bread, eggs, hydrogenated fats, pasta, sugar, and sweets. However, there is insufficient data to determine if the cultural variables of Iranians can affect their eating pattern.

As far as the researchers of this study investigated, the present study is the first to analyze the relationship between MIND diet score, mental health, and cardiometabolic risk factors among individuals with obesity. Also, the analysis was performed in a relatively acceptable sample size, dietary intake was assessed by a validated FFQ, and the role of possible confounders was controlled by appropriate statistical methods. However, a number of limitations of the study must also be acknowledged. First, the study’s cross-sectional design makes it difficult to obtain a causal association between study parameters. Second, although we adjusted for the confounders, the residual confounders could not be removed. Third, since the consumption of wine is legally prohibited in Iran [97], the related data might have a potential bias affecting data reliability. Hence, we did not collect any data on wine consumption. In conclusion, higher tertiles of the MIND diet score were associated with increased insulin sensitivity and reduced stress among individuals with obesity significantly. Further well-designed longitudinal studies are needed to elucidate the causal associations better and confirm our results.

## Supplementary Information

**Additional file 1.**

## Data Availability

The datasets generated and/or analyzed during the current study are not publicly available due privacy and ethical considerations, but can be available from the corresponding author on reasonable request.
