# Supplementary material for: Evaluating the association between the Mediterranean-DASH Intervention for Neurodegenerative Delay (MIND) diet, mental health, and cardio-metabolic risk factors among individuals with obesity
Source: BMC Endocr Disord. 2023 Feb 2;23:29. doi: 10.1186/s12902-023-01284-8 (PMC9893576; doi:10.1186/s12902-023-01284-8)
Supplement: Supplementary file 1 — Additional file 1. [file 12902_2023_1284_MOESM1_ESM.docx]

**Table S1.** Crude and multivariable adjusted ORs and 95% CIs for cardio-metabolic variables across tertiles of non-berry fruits

**P*<0.05; ^a^ Model I: adjusted for age, sex, BMI, and physical activity; ^b^ Model II: additionally adjusted for socioeconomic status and energy intake.

Abbreviations; SBP, systolic blood pressure; DBP, diastolic blood pressure; FBS, fasting blood sugar; TC, total cholesterol; HDL, high density lipoprotein cholesterol; LDL, low density lipoprotein cholesterol; TG, triglyceride; HOMA-IR, homeostatic model assessment for insulin resistance; QUICKI, quantitative insulin-sensitivity check index; AgRP: Agouti-Related Protein; α-MSH: α-Melanocyte-stimulating hormone.

| **Variables** | **Tertiles of non-berry fruits** | | | | | | | | | | | |
| --- | --- | --- | --- | --- | --- | --- | --- | --- | --- | --- | --- | --- |
|  | | **Crude ORs (95% CI)** | | |  | **Model I^a^ ORs (95% CI)** | | |  | **Model II^b^ ORs (95% CI)** | | |
|  | | **1^st^ )n=113 )** | **2^nd^ (n=113)** | **3^rd^ (n=113)** |  | **1^st^ )n=113 )** | **2^nd^ (n=113)** | **3^rd^ (n=113)** |  | **1^st^ )n=113 )** | **2^nd^ (n=113)** | **3^rd^ (n=113)** |
| SBP (mmHg) | | 1 | **0.973 (0.956-0.990) *** | 0.984 (0.968-1.001) |  | 1 | 0.999 (0.970-1.028) | 0.990 (0.959-1.022) |  | 1 | 0.999 (0.970-1.028) | 0.986 (0.953-1.020) |
| DBP (mmHg) | | 1 | **0.954 (0.930-0.978) *** | 0.980 (0.956-1.004) |  | 1 | **0.960 (0.926-0.996) *** | 0.968 (0.932-1.006) |  | 1 | **0.961 (0.927-0.997) *** | 0.967 (0.928-1.007) |
| FBS (mg/dl) | | 1 | 1.007 (0.993-1.021) | 0.997 (0.980-1.013) |  | 1 | 1.013 (0.995-1.032) | 1.005 (0.983-1.027) |  | 1 | 1.013 (0.995-1.032) | 1.004 (0.982-1.027) |
| TC (mg/dL) | | 1 | **0.993 (0.986-0.999) *** | 0.998 (0.991-1.005) |  | 1 | 0.993 (0.982-1.005) | 0.997 (0.985-1.010) |  | 1 | 0.994 (0.982-1.005) | 0.998 (0.986-1.011) |
| LDL-C (mg/dL) | | 1 | **0.992 (0.983-0.999) *** | 1.000 (0.992-1.008) |  | 1 | 0.992 (0.979-1.004) | 0.996 (0.983-1.010) |  | 1 | 0.992 (0.980-1.005) | 0.997 (0.983-1.011) |
| HDL-C (mg/dL) | | 1 | 1.011 (0.983-1.039) | 1.017 (0.989-1.046) |  | 1 | 1.022 (0.977-1.068) | 1.030 (0.982-1.080) |  | 1 | 1.020 (0.976-1.066) | 1.028 (0.979-1.080) |
| TG (mg/dL) | | 1 | **0.997 (0.993-0.999) *** | 0.999 (0.996-1.001) |  | 1 | 0.998 (0.992-1.004) | 0.998 (0.991-1.005) |  | 1 | 0.998 (0.992-1.004) | 0.998 (0.991-1.005) |
| Insulin (µIU/mL) | | 1 | 0.004 (0.982-1.027) | 1.000 (0.976-1.025) |  | 1 | 1.039 (0.992-1.088) | 1.041 (0.991-1.093) |  | 1 | 1.041 (0.994-1.091) | 1.050 (0.998-1.105) |
| HOMA-IR | | 1 | 1.041 (0.945-1.147) | 1.003 (0.901-1.117) |  | 1 | 1.210 (1.009-1.450) | 1.180 (0.975-1.427) |  | 1 | 1.217 (1.012-1.465) | 1.211 (0.993-1.476) |
| QUICKI | | 1 | 1.357 (0.692-1.890) | 1.550 (1.422-1.783) |  | 1 | 1.439 (0.518-1.796) | 1.806 (1.779-1.833) |  | 1 | 1.393 (0.931-1.811) | 1.223 (0.824-1.713) |

**Table S2.** Crude and multivariable adjusted ORs and 95% CIs for cardio-metabolic variables across tertiles of low-fat dairy

**P*<0.05; ^a^ Model I: adjusted for age, sex, BMI, physical activity, socioeconomic status, and education level; ^b^ Model II: additionally adjusted for energy intake.

Abbreviations; SBP, systolic blood pressure; DBP, diastolic blood pressure; FBS, fasting blood sugar; TC, total cholesterol; HDL, high density lipoprotein cholesterol; LDL, low density lipoprotein cholesterol; TG, triglyceride; HOMA-IR, homeostatic model assessment for insulin resistance; QUICKI, quantitative insulin-sensitivity check index; AgRP: Agouti-Related Protein; α-MSH: α-Melanocyte-stimulating hormone.

| **Variables** | **Tertiles of low-fat dairy** | | | | | | | | | | | |
| --- | --- | --- | --- | --- | --- | --- | --- | --- | --- | --- | --- | --- |
|  | | **Crude ORs (95% CI)** | | |  | **Model I^a^ ORs (95% CI)** | | |  | **Model II^b^ ORs (95% CI)** | | |
|  | | **1^st^ )n=113 )** | **2^nd^ (n=113)** | **3^rd^ (n=113)** |  | **1^st^ )n=113 )** | **2^nd^ (n=113)** | **3^rd^ (n=113)** |  | **1^st^ )n=113 )** | **2^nd^ (n=113)** | **3^rd^ (n=113)** |
| SBP (mmHg) | | 1 | 1.003 (0.987-1.019) | 1.009 (0.993-1.026) |  | 1 | 1.019 (0.990-1.048) | 1.018 (0.988-1.049) |  | 1 | 1.018 (0.989-1.048) | 1.018 (0.986-1.050) |
| DBP (mmHg) | | 1 | 1.005 (0.983-1.027) | 1.012 (0.989-1.035) |  | 1 | 1.009 (0.977-1.043) | 1.006 (0.971-1.042) |  | 1 | 1.010 (0.977-1.043) | 1.005 (0.969-1.043) |
| FBS (mg/dl) | | 1 | 1.007 (0.990-1.024) | 1.013 (0.997-1.029) |  | 1 | 1.000 (0.978-1.023) | 1.013 (0.993-1.034) |  | 1 | 1.000 (0.978-1.022) | 1.013 (0.992-1.033) |
| TC (mg/dL) | | 1 | 1.003 (0.995-1.010) | 1.000 (0.993-1.007) |  | 1 | 0.999 (0.989-1.010) | 0.998 (0.987-1.010) |  | 1 | 1.000 (0.989-1.010) | 0.998 (0.987-1.010) |
| LDL-C (mg/dL) | | 1 | 1.004 (0.995-1.012) | 1.003 (0.994-1.011) |  | 1 | 1.001 (0.989-1.012) | 0.997 (0.985-1.009) |  | 1 | 1.001 (0.990-1.013) | 0.997 (0.984-1.010) |
| HDL-C (mg/dL) | | 1 | 0.980 (0.953-1.007) | 0.993 (0.967-1.021) |  | 1 | 0.960 (0.921-1.001) | 0.986 (0.945-1.029) |  | 1 | 0.961 (0.922-1.002) | 0.985 (0.942-1.030) |
| TG (mg/dL) | | 1 | 0.998 (0.995-1.001) | 1.001 (0.998-1.004) |  | 1 | 1.003 (0.996-1.009) | 1.003 (0.997-1.010) |  | 1 | 1.003 (0.996-1.009) | 1.003 (0.996-1.010) |
| Insulin (µIU/mL) | | 1 | 0.997 (0.975-1.019) | 0.993 (0.969-1.017) |  | 1 | 1.000 (0.963-1.039) | 0.997 (0.956-1.039) |  | 1 | 1.001 (0.963-1.040) | 1.001 (0.958-1.045) |
| HOMA-IR | | 1 | 0.990 (0.900-1.090) | 1.003 (0.914-1.101) |  | 1 | 1.002 (0.866-1.159) | 1.049 (0.905-1.216) |  | 1 | 1.004 (0.867-1.163) | 1.065 (0.913-1.242) |
| QUICKI | | 1 | 1.002 (0.589-3.84) | 1.010 (0.709-1.387) |  | 1 | 1.038 (0.726-4.622) | 1.142 (0.851-1.99) |  | 1 | 1.032 (0.787-4.201) | 1.162 (0.763-2.029) |

**Table S3.** Crude and multivariable adjusted ORs and 95% CIs for cardio-metabolic variables across tertiles of high-fat dairy

| **Variables** | **Tertiles of high-fat dairy** | | | | | | | | | | | |
| --- | --- | --- | --- | --- | --- | --- | --- | --- | --- | --- | --- | --- |
|  | | **Crude ORs (95% CI)** | | |  | **Model I^a^ ORs (95% CI)** | | |  | **Model II^b^ ORs (95% CI)** | | |
|  | | **1^st^ )n=113 )** | **2^nd^ (n=113)** | **3^rd^ (n=113)** |  | **1^st^ )n=113 )** | **2^nd^ (n=113)** | **3^rd^ (n=113)** |  | **1^st^ )n=113 )** | **2^nd^ (n=113)** | **3^rd^ (n=113)** |
| SBP (mmHg) | | 1 | 0.990 (0.974-1.006) | 0.997 (0.981-1.013) |  | 1 | 0.999 (0.965-1.020) | 0.993 (0.962-1.024) |  | 1 | 0.992 (0.965-1.020) | 0.990 (0.958-1.023) |
| DBP (mmHg) | | 1 | **0.977 (0.955-0.999) *** | 0.990 (0.968-1.013) |  | 1 | 0.979 (0.948-1.012) | 0.982 (0.946-1.019) |  | 1 | 0.981 (0.949-1.014) | 0.981 (0.944-1.020) |
| FBS (mg/dl) | | 1 | 1.003 (0.989-1.016) | 1.000 (0.986-1.015) |  | 1 | 1.002 (0.982-1.024) | 1.011 (0.991-1.031) |  | 1 | 1.001 (0.981-1.022) | 1.010 (0.990-1.031) |
| TC (mg/dL) | | 1 | 0.997 (0.990-1.004) | 0.999 (0.992-1.006) |  | 1 | 0.996 (0.985-1.006) | 0.998 (0.986-1.010) |  | 1 | 0.996 (0.986-1.007) | 0.999 (0.986-1.011) |
| LDL-C (mg/dL) | | 1 | 0.996 (0.988-1.005) | 0.998 (0.990-1.006) |  | 1 | 0.997 (0.986-1.009) | 0.999 (0.987-1.012) |  | 1 | 0.998 (0.987-1.010) | 1..001 (0.987-1.014) |
| HDL-C (mg/dL) | | 1 | **1.029 (1.001-1.058) *** | 0.985 (0.957-1.014) |  | 1 | 1.022 (0.981-1.064) | 0.969 (0.924-1.016) |  | 1 | 1.019 (0.978-1.062) | 0.962 (0.915-1.011) |
| TG (mg/dL) | | 1 | 0.998 (0.995-1.002) | 1.002 (0.999-1.004) |  | 1 | **0.993 (0.986-0.999)*** | 1.001 (0.994-1.007) |  | 1 | **0.993 (0.986-0.999)*** | 1.001 (0.994-1.007) |
| Insulin (µIU/mL) | | 1 | 1.019 (0.992-1.047) | 1.008 (0.979-1.038) |  | 1 | 1.007 (0.969-1.047) | 1.012 (0.969-1.057) |  | 1 | 1.008 (0.969-1.049) | 1.018 (0.973-1.065) |
| HOMA-IR | | 1 | 1.055 (0.955-1.165) | 1.014 (0.908-1.133) |  | 1 | 1.010 (0.870-1.173) | 1.068 (0.911-1.252) |  | 1 | 1.011 (0.869-1.176) | 1.085 (0.921-1.279) |
| QUICKI | | 1 | 1.232 (0.627-4.01) | 1.017 (0.708-1.453) |  | 1 | 1.211 (0.703-4.76) | 1.312 (0.794-2.01) |  | 1 | 1.024 (0.741-4.001) | 1.137 (0.743-2.064) |

**P*<0.05; ^a^ Model I: adjusted for age, sex, BMI, physical activity, socioeconomic status, and education level; ^b^ Model II: additionally adjusted for energy intake.

Abbreviations; SBP, systolic blood pressure; DBP, diastolic blood pressure; FBS, fasting blood sugar; TC, total cholesterol; LDL-C, low density lipoprotein cholesterol; HDL-C, high density lipoprotein cholesterol; TG, triglyceride; HOMA-IR, homeostatic model assessment for insulin resistance; QUICKI, quantitative insulin-sensitivity check index; AgRP: Agouti-Related Protein; α-MSH: α-Melanocyte-stimulating hormone.
